# Case Report: The Complete Remission of a Mixed Germ Cell Tumor With Somatic Type Malignancy of Sarcoma Type With a GCT-Oriented Therapy: Clinical Findings and Genomic Profiling

**DOI:** 10.3389/fonc.2021.633543

**Published:** 2021-03-16

**Authors:** Maria A. Pantaleo, Marcella Mandruzzato, Valentina Indio, Milena Urbini, Margherita Nannini, Lidia Gatto, Angela Schipani, Michelangelo Fiorentino, Tania Franceschini, Valentina Ambrosini, Valerio Di Scioscio, Maristella Saponara, Manuela Ianni, Sergio Concetti, Annalisa Altimari, Andrea Ardizzoni, Annalisa Astolfi

**Affiliations:** ^1^Division of Oncology, IRCSS Azienda Ospedaliero Universitaria di Bologna, Bologna, Italy; ^2^“Giorgio Prodi Cancer Research Center” and Department of Experimental, Diagnostic and Specialized Medicine, University of Bologna, Bologna, Italy; ^3^Biosciences Laboratory, Istituto Scientifico Romagnolo per lo Studio e la Cura dei Tumori IRCCS, Meldola, Italy; ^4^Metropolitan Department of Pathology, University of Bologna, Bologna, Italy; ^5^Division of Nuclear Medicine Azienda Ospedaliero Universitaria di Bologna, Bologna, Italy; ^6^Division of Radiology Azienda Ospedaliero Universitaria di Bologna, Bologna, Italy; ^7^UOC Urologia, Azienda Unità Sanitaria Locale (AUSL), Bologna, Italy; ^8^Division of Laboratory of Oncologic Molecular Pathology, Azienda Ospedaliero Universitaria di Bologna, Bologna, Italy; ^9^Department of Translational Medicine, University of Ferrara, Ferrara, Italy

**Keywords:** teratoma with malignant transformation, germ cell tumor, sarcoma, PEB, RNA-sequencing, exome sequencing

## Abstract

Somatic malignant transformation in a germ cell tumor (GCT) is the development of non-germ malignancies; much of available literature refers to teratoma with malignant transformation (TMT). There are various transformation histologies such as sarcoma, adenocarcinoma, primitive neuroectodermal tumors, and more rarely carcinoid tumors, hemangioendothelioma, lymphoma, or nephroblastoma. The treatments of these entities include surgery and/or chemotherapy. A standard approach in choosing chemotherapy in TMT cases has not yet been established. Many authors suggest using chemotherapeutic agents based on the transformed histology, while others recommend GCT-oriented therapy combined with surgery as the primary treatment, reserving histology-driven chemotherapies for metastatic relapse. We report the clinical findings and the genomic profile of a mixed GCT case with somatic-type malignancy of sarcoma type. We achieved a complete radiological response with GCT-oriented chemotherapy performed as salvage therapy after sarcoma-histology therapy. In addition, molecular profiles with RNA-sequencing and exome sequencing analyses of the primary tumor and the tumor with somatic-type malignancy of sarcoma type were explored.

## Introduction

Somatic malignant transformation in a germ cell tumor (GCT) is the development of non-germ cell malignancies. Much of the available literature on this phenomenon refers to teratoma with malignant transformation (TMT), which is rare and accounts for ~3–6.6% of GCTs (1–4). Many case series of secondary malignant transformation in GCT have been reported. The first of eleven cases was described by Ulbright in 1984, when, for the first time, the idea was theorized that the malignant elements could derive from teratomatous foci, since eight of nine cases available for review had teratoma in the primary tumor and teratoma was found in all subsequently recurring lesions (5–10). The etiological hypothesis of this malignancy is different: given the remarkable chemo-sensitivity of GCTs, chemotherapy could eliminate the bulk of GCTs, thus exposing the non-GCT, chemo-resistant malignant components (5), or TMT could derive from histological transformation due to pluripotency of GCT components. The commonest histology of malignant transformation is sarcoma, with rhabdomyosarcoma being the most frequent subtype (5, 10–12), followed by adenocarcinoma, primitive neuroectodermal tumor, and in rare cases, even carcinoid tumors, hemangioendothelioma, lymphoma, or nephroblastoma (5, 13, 14).

Chemotherapeutic regimens commonly fail to eradicate teratomas with somatic-type malignancy (8, 15); therefore, the strongest prognostic factor remains the tumor burden and the consequence is to perform radical tumor removal. The three most important favorable prognostic factors, independent by stage and risk category according to the International Germ Cell Cancer Collaborative Group (IGCCCG) classification, are as follows: non-primitive neuroectodermal tumor histology, primary gonadal tumor site, and low number of chemotherapy regimens administered before TMT diagnosis (16). Although the rarity of GCTs with malignant transformation have not permitted the generation of a strong body of evidence, it is agreed that a surgical approach with complete resection is the standard treatment, independent of disease stage (5, 17, 18). However, there is not yet a clear standard of care for target chemotherapies in these neoplasms because there are only a few retrospective case series with different somatic components and heterogeneous results (13–15, 19–22). Many authors suggested using chemotherapeutic agents based on the transformed histology (13, 19), while others advocated GCT-oriented therapy combined with timely surgery as the primary treatment, reserving histology-driven chemotherapies for relapse (14, 20).

In this intricate setting, we report a case of a complete response in a patient with of high-grade soft tissue sarcoma originating from a mixed GCT (a mixed GCT with somatic-type malignancy of sarcoma type) treated with GCT-oriented therapy. This case report further enlarges the body of evidence and may help clinicians in the treatment choice in this setting.

## Case Description

### Patient Timeline

In August 2015, a 50-year-old man developed an increase in size of the right testicle without additional clinical findings. Laboratory testing showed normal serum levels of alpha-fetoprotein and carcinoembryonic antigen (CEA), and high levels of lactate dehydrogenase (LDH) (375 U/L) and beta-human chorionic gonadotrophin (B-hCG) (1.9 mU/mL). The testicular ultrasound showed a multiple hypo-echoic area suspected to be a testicular tumor. The computed tomography (CT) scan was negative for metastatic lesions.

The patient underwent a right radical orchiectomy for the testicular mass; histological examination was compatible with pure seminoma. The tumor was completely resected, margins were negative, and the funiculus was free of neoplasia. The tumor was classified as pT2, N0, MX, stage I. The absence of lymph node involvement or distant metastases and the normalization of B-hCG after surgery suggested an active surveillance approach.

**Figure 1:**
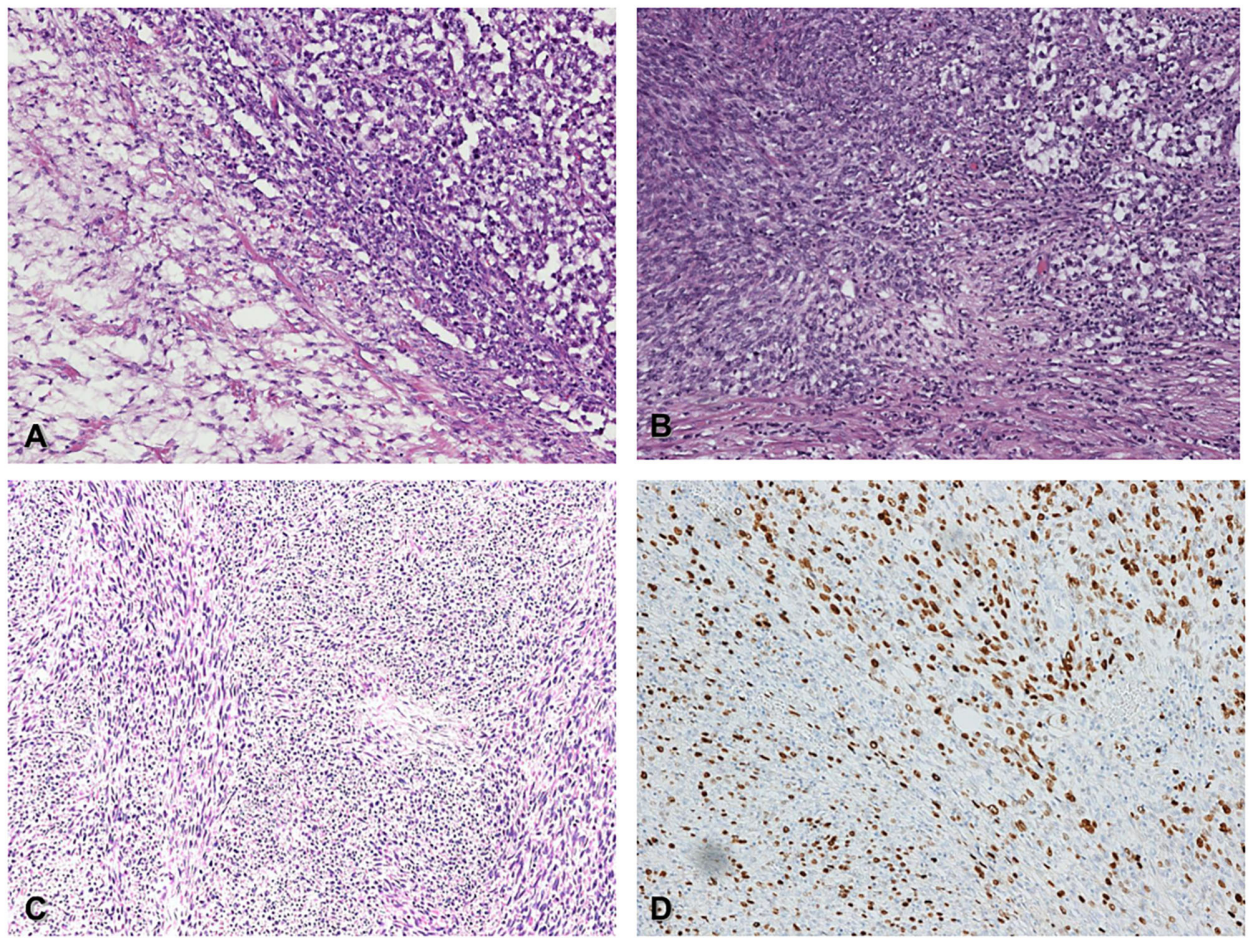
Histological features of germ-cell tumor (seminoma) with somatic malignant transformation (spindle cell sarcoma) **(A)** Classic seminoma (upper right corner) associated with spindle cell sarcoma (lower left corner), (H&E 200x). **(B)** *in situ* germ cell neoplasia (upper right corner) admixed with somatic spindle cell sarcoma (lower left corner), (H&E 200x). **(C)** Metastatic spindle cell sarcoma within the spermatic cord (H&E 200x). **(D)** The same field of picture **C** shows high proliferative Ki67 index (DAB 200x).

During the follow up, in September 2016, the patient was in a well condition when he suddenly developed an inguinal mass without associated symptoms (no pain, no urological symptoms). The CT showed an inguinal-scrotal mass 15 × 7 cm in size, which was suspected as recurrence of the tumor; no additional suspected lesions were reported. Serum levels of alpha-fetoprotein, CEA, and B-hCG were in the normal range, whereas LDH was slightly elevated from the normal range (260 U/L). Thus, the patient underwent removal of the lesion and histological examination revealed a high-grade unclassified soft tissue sarcoma with spindle cells. The patient was then referred to the Sarcoma Study Group of our institution and, in consideration of the oncological history, a revision of the previous histological exams was performed. The comparison between the two histological samples demonstrated that the primary testicular neoplasm was a rare case of mixed GCT composed of a seminomatous and a teratomatous component. A high-grade malignant mesenchymal neoplasm with spindle cells was also found to be present, which probably originated from a malignant somatic transformation of the teratomatous component (Figures 1A,B).

The primary testicular tumor, a germinal tumor with prevalent seminoma component admixed with a spindle cell sarcoma component, is a somatic transformation of a pre-existent immature teratoma. The seminoma component was immunoreactive for CD117 and OCT3/4. The sarcoma component was negative for OCT3/4, as a demonstration of the somatic type malignancy. The spindle cell sarcoma was negative for wide-spectrum cytokeratins, MDM2, smooth muscle actin, S-100, and SOX-10, with the immunoprofile of a fibrosarcoma. The coexistence of a pure seminoma component with the somatic type sarcoma was quite peculiar. The microscopic morphology of the recurring inguinal lesion was the testicular high-grade sarcomatous component of the first testicular mass, according to the hypothesis that the inguinal lesion was a recurrence of the mesenchymal component (Figures 1C,D). The histology of the groin metastasis resembled completely the features and the immunoprofile of the primary sarcoma component.

**Figure 2:**
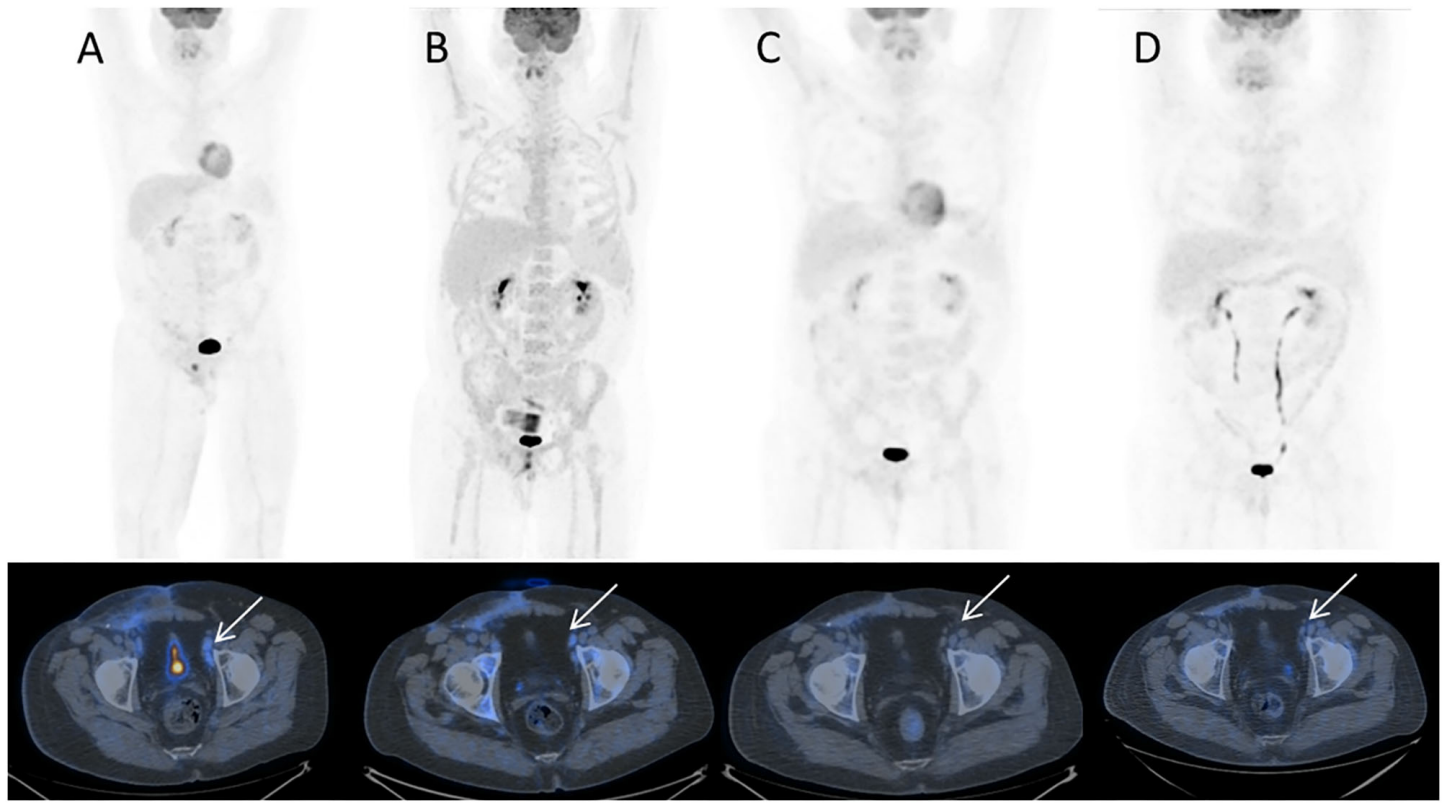
18F-FDG PET/CT MIP and corresponding trans-axial images obtained at staging. **(A)** December 2016 before sarcoma therapy; **(B)** March 2017 after sarcoma therapy; **(C)** May 2017 early evaluation after two cycles of chemotherapy for GCT testicular cancer; **(D)** July 2017 at completion of GCT testicular cancer chemotherapy. Hypermetabolic left external iliac nodes (**A**, white arrow) show only slight decrease of uptake after sarcoma chemotherapy (**B**, white arrow) while they turn FDG-negative after chemotherapy for testicular cancer, respectively, after 2 cycles (**C**, white arrow) and 4 cycles (**D**, white arrow). Note bone marrow rebound after chemotherapy in **B**.

**Figure 3:**
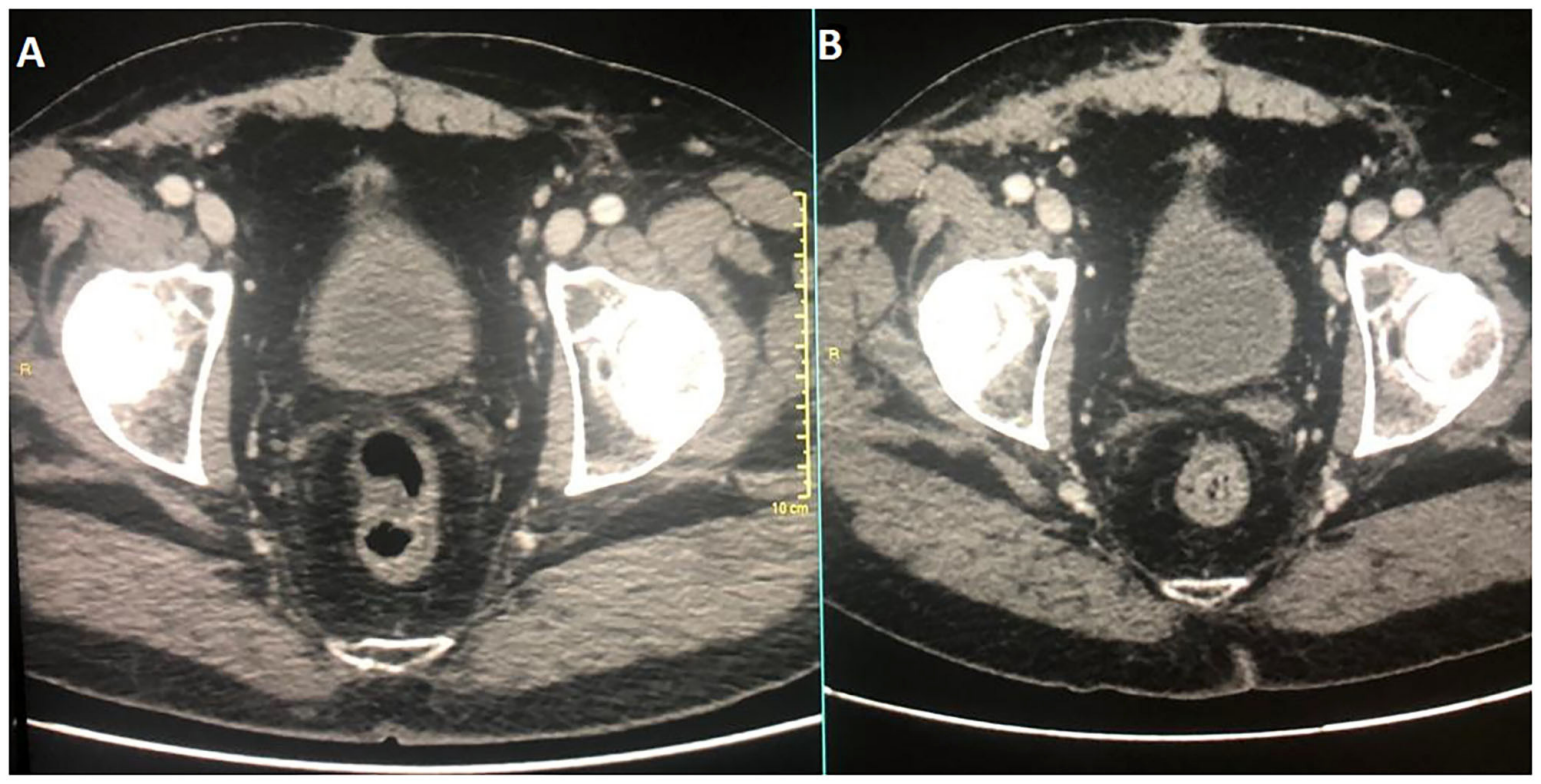
Diagnostic CT corresponding images at diagnosis and at therapy completion in **(A,B)**, respectively.

**Figure 4:**
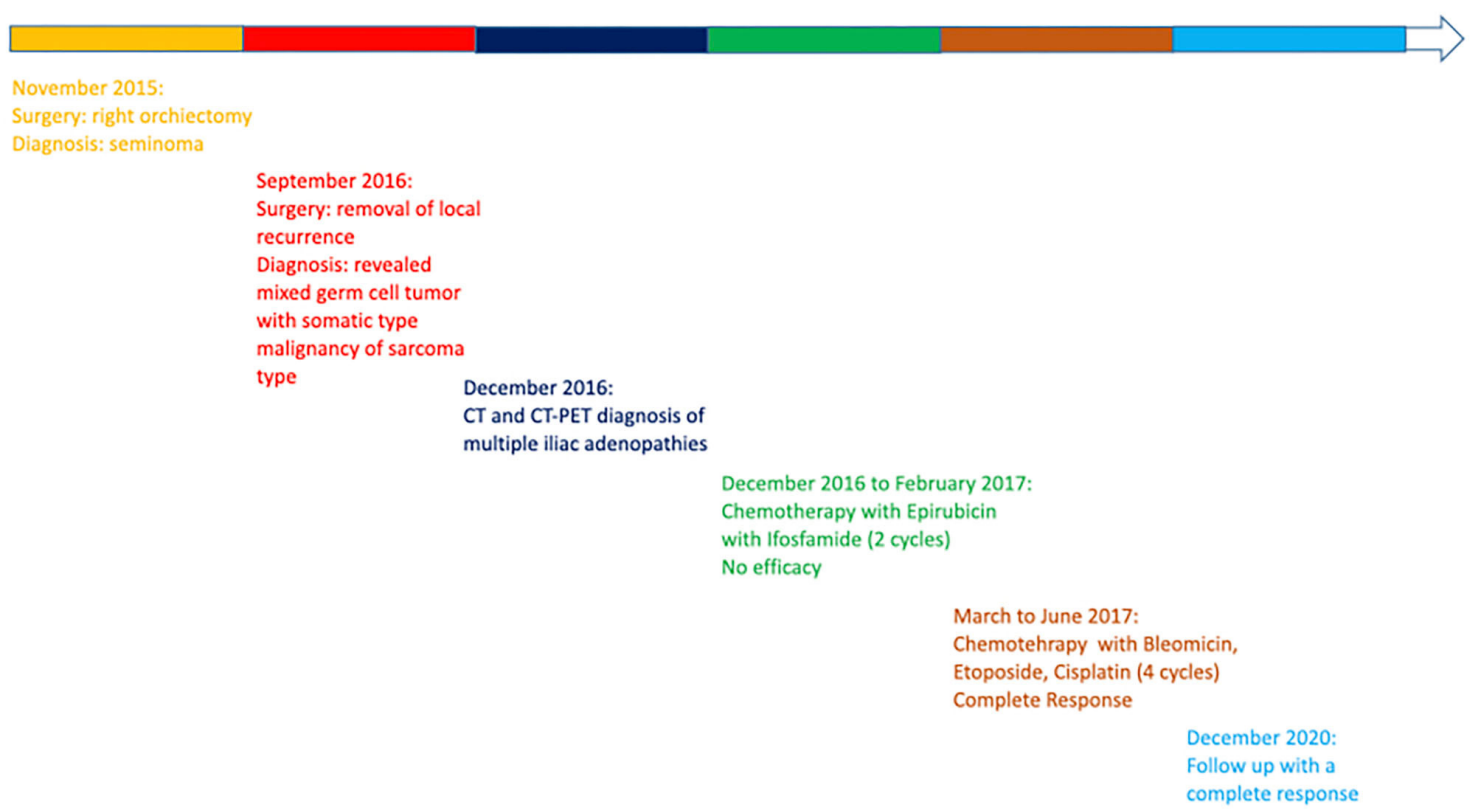
Figure showcasing a timeline with relevant data of the clinical history of patient.

In December 2016, the patient underwent imaging with CT- and FDG-Positron Emission Tomography (PET) for suspicion of a new relapse. The FDG-PET scan showed high glucose uptake in multiple iliac adenopathies [max standardized uptake value (SUV) = 4.4; Figure 2A]. A CT scan was performed 1 month after surgery, which showed a liquid mass 6.7 cm in diameter in the right inguinal cavity and multiple bilateral iliac adenopathies (Figure 3A). Considering the metastatic stage of the disease, the patient was directed to receive systemic therapy. The choice of the treatment protocol was driven by the sarcomatous histology; first-line treatment with epirubicin and ifosfamide was performed from December 2016 to February 2017. The subsequent abdominal-chest CT scan revaluation showed a substantial stabilization of the disease; however, FDG-PET revealed only a mild reduction in size and in glucose-uptake of the multiple iliac adenopathies (SUV max 3.7 vs. 4.4; Figure 2B). In consideration of the not-entirely satisfactory response to chemotherapy oriented by the exclusive presence of sarcomatous cells, we decided to switch toward a GCT-oriented therapy. Thus, from March to June 2017, the patient was treated with a total of four cycles of BEP therapy (Bleomicin, Etoposide, Cisplatin), with good tolerance. An early revaluation of the disease by FDG-PET after two cycles of therapy revealed a complete response of the disease with total disappearance of the previous uptakes (Figure 2C). A complete response was then confirmed by CT-PET (Figure 2D) and a chest-abdominal CT scan (Figure 3B), which was performed after the end of the treatment. The patient started a surveillance program with a CT scan and CT-PET every 4 months. He remains disease-free at last follow-up visit, 40 months after complete remission, and tumoral markers have remained negative. A timeline with the relevant data of this patient is shown in Figure 4.

### RNA-seq and Exome Sequencing of the Tumors

Given these clinical findings, we decided to characterize both the primary tumor and the recurrence in the sarcoma with whole transcriptome sequencing (RNA-seq) and exome sequencing to explore the molecular backgrounds and to further investigate any correlation with clinical course and response.

RNA-seq was done on Nextseq500 Illumina platform. RNA was extracted from the manually macrodissected tumor tissue from the FFPE slides using RecoverAll Total Nucleic Acid Isolation Kit (Thermo Fisher Scientific). Tumor tissue enrichment was at least 70%. cDNA libraries were obtained using TruSeq RNA Exome kit (Illumina), following the manufacturer's instructions. Paired-end reads were mapped on the human Reference Genome hg38 with the alignment algorithm STAR. Transcript abundance was quantified with the Python package *Htseq-count* and normalized as counts per million (cpm) adopting the R-bioconductor package *edgeR*.

**Figure 5:**
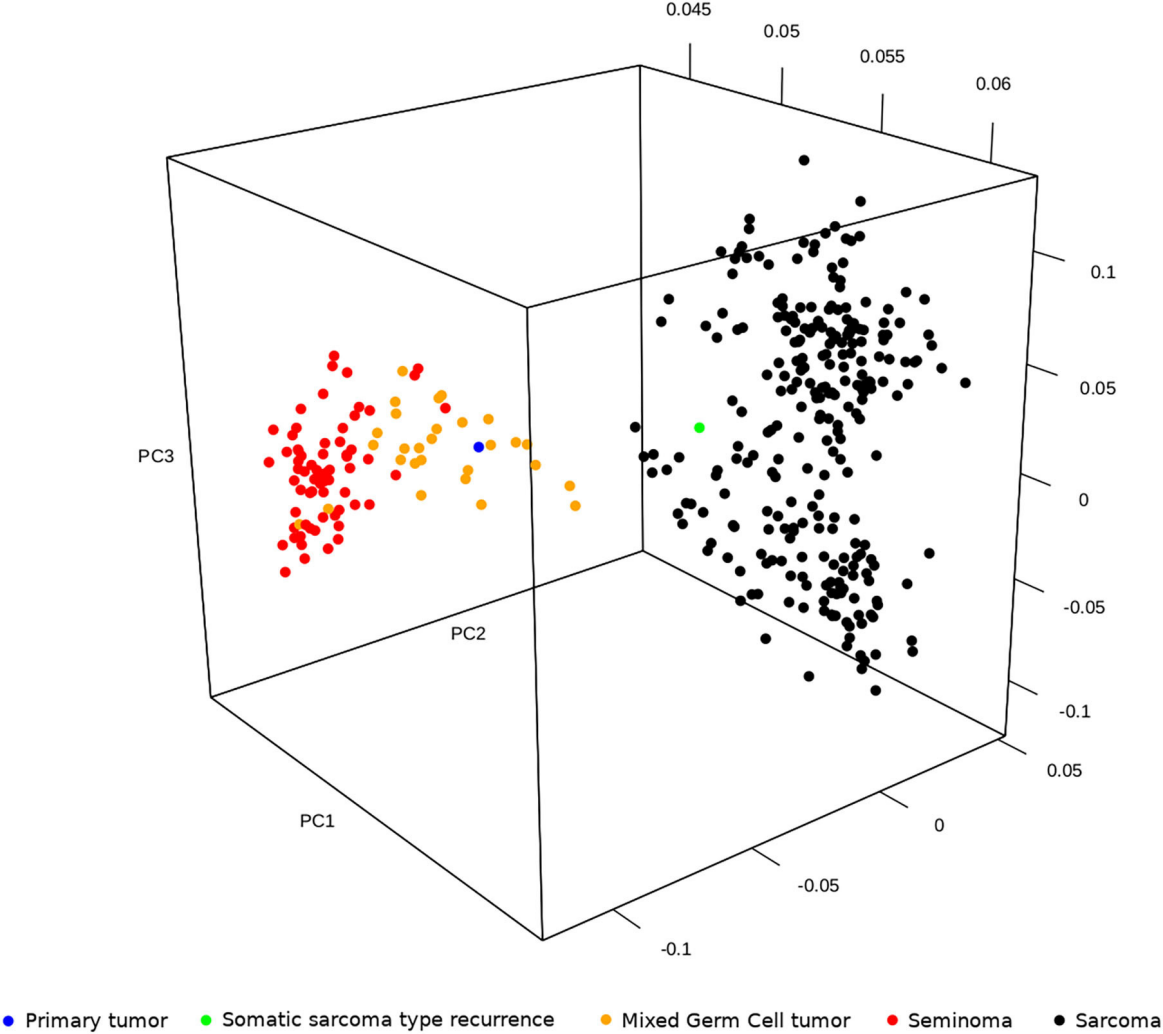
Principal component analysis of seminoma, mixed germ cell tumors and sarcoma publicly available in The Cancer Genome Atlas project (TCGA) and our primary tumor and relapse in sarcoma.

Primary tumor and TMT expression profiles were compared with transcriptome data of testicular GCTs and sarcoma publicly available in The Cancer Genome Atlas project (TCGA). In particular, as testicular neoplasm, we included the subset of 94 tumors with labels “Germ Cell Neoplasms” as Disease Type, “Testis” as Primary Site and “Seminoma” or “Mixed germ cell tumor” as Primary Diagnosis (66 and 28 samples, respectively); as sarcoma, we included 259 samples with the label “Soft tissue tumors and sarcoma” as Disease Type. The principal component analysis of seminoma, mixed GCTs, and sarcoma (from TCGA) and our primary tumor and recurrece samples was performed adopting the R package *prcomp*. The results showed that the expression profile of sarcoma cases was distinctly separate from testicular tumors, which, in turn, are grouped in seminoma and mixed GCTs. Moreover, including our case, the analysis highlighted that the primary testicular tumor is closer to a mixed GCT and that the TMT is more similar to sarcoma (Figure 5). These observations, which clearly agree with the histological diagnosis, suggested that the complete response of TMT after the PEB protocol cannot be explained by the global expression profile itself. It is reasonable to hypothesize that specific gene signatures or expression patterns could be related to chemosensitivity; however, unfortunately, we are not able to perform additional statistical analysis on a single patient.

For a more in-depth view we also performed targeted exome sequencing with the aim to study genetic alterations in both the primary and TMT in sarcoma samples. Targeted sequencing and data analysis were performed by the Foundation One CDx test, according the standard procedures of the Service. The test detects substitutions, indels, and copy number alterations (CNAs) in 324 genes, and gene rearrangements in 34 selected genes. The results revealed very few mutational events (Supplementary Table 1) that were shared between the diagnosis and relapse samples. Concordantly, the tumor mutational burden (TMB) was low (3–4 mutations/Mb), and microsatellite status was stable. The most relevant events were the amplification of 12p12.1, overlapping KRAS, and a region at chr4q12, encompassing KIT, PDGFRA, and KDR genes. All the other mutational events shared between the two samples were variants of unknown significance (VUS). The only novel acquisition of the sarcomatous relapse was a rearrangement of NF1 at exon 54. These results confirm the genomic stability of the tumor, which was maintained in the TMT relapse.

## Discussion

We report a case of a complete radiological response in a patient with a mixed GCT with somatic-type malignancy of sarcoma type, which was obtained by GCT-oriented chemotherapy as a salvage therapy after sarcoma histology therapy.

The non-germ cell malignant transformation from a GCT is rare but is well described in many papers, most of which refer to TMT. Due to the rarity of disease, little information is available on the pathogenesis, prognosis, and treatment. The clinical history of the patient revealed interesting clinical findings. In fact, he experienced an unusual and aggressive local recurrence even though the primary tumor was treated with complete surgery at initial diagnosis. This uncommon oncological behavior, even if not proven, may be associated with the aggressively transformed component of sarcoma. The patient went on to develop metastatic lymph nodes, which were firstly treated with sarcoma-oriented therapy without success and then with GCT-oriented chemotherapy with complete remission.

A consensus on the standard approach in choosing chemotherapy in cases with somatic transformation in GCTs has not yet been reached. Many authors have suggested using chemotherapeutic agents based on the transformed histology (13, 19), while others suggested GCT-oriented therapy combined with surgery as the primary treatment, reserving histology-driven chemotherapies for metastatic relapse (14, 20). However, due to the few retrospective series published combined with the different somatic components and the different chemotherapeutic approaches used, the results are heterogeneous and not conclusive. To our knowledge, only a few cases have been reported with a complete remission after GCT therapy in some case series (14, 20); however, they have never been described in detail.

In the present case report, our attention is mainly focused on somatic transformation in sarcoma. Soft tissue sarcoma is a highly heterogeneous tumor and occurs in complex oncological settings. More than 50 subtypes of soft tissue sarcoma have been described, all with different clinical presentations, prognoses and treatments. Currently, several chemotherapeutic agents are available for the treatments of advanced sarcoma. Three standard lines of therapy have been approved including doxorubicin with or without ifosfamide, which are considered the first choice in clinical practice, and also gemcitabine, docetaxel, trabectidin, pazopanib, and temozolomide or dacarbazine (23). Moreover, although very rare, some subtypes of sarcoma present with genomic aberration such as the anaplastic lymphoma kinase gene (ALK gene) rearrangements in inflammatory myofibroblastic tumor (IMT) or NTRK gene rearrangements, and others for which specific inhibitors have recently been developed as alternative treatments.

In our case, the standard therapy based on the anthracycline combination did not result in a satisfactory response, which even in absence of serum markers increase directed our choice to GCT-oriented therapy with a successful response. The molecular data, especially the gene expression profile, showed that the primary tumor clusters with testicular tumors while the recurrence clusters with sarcoma. Despite the morphology and the molecular landscape suggest closeness to sarcoma, the recurrence showed a biological response to chemotherapy that followed the primary histology origin. Thus, molecular alterations identified in our case were shared between GCT and transformed sarcoma recurrence samples indicating the genomic stability of these tumors. Amplification of 12p12.1, which is commonly found in testicular GCT and is a putative driver gene of the disease (24), was maintained between the two tumors in our case. However, this alteration is not considered as predictive of survival or response to therapy (25). Conversely, sensitivity to cisplatin was correlated to a higher apoptotic response and reduced ability to repair cisplatin-induced DNA damage, while resistance was associated with inactivation of the TP53 pathway, defective mismatch repair (MMR), and microsatellite instability (26, 27). Consistent with these data, in our case, both primary GCT and sarcoma-type recurrence showed a low mutational burden. Furthermore, no mutations were detected in TP53 pathway, suggesting a cisplatin-sensitive phenotype.

The clinical and molecular findings of our case may suggest that clinicians who administer GCT for malignant transformation in sarcoma cases need to evaluate early the efficacy of the therapy whether it is GCT- or sarcoma-histology-oriented. This suggestion may be useful because the low number of chemotherapy regimens administered represents a prognostic factor for disease control. Moreover, the sarcoma-oriented therapy benefits at least three lines of different chemotherapy regimens, and consequently, the GCT-oriented therapy could be administered too late for patients who develop a rapidly progressive disease and worsening of clinical conditions. This was a successful case treated with GCT-oriented chemotherapy instead of sarcoma histology therapy. Since it is not sufficient to outline the guidelines in clinical practice, it may be necessary to recommend that the clinicians pay attention to the re-evaluation of the first therapy efficacy. Moreover, this approach could not be totally applied to other histologic somatic transformations.

As an additional finding, we highlight the role of FDG-PET in the early prediction of a therapeutic response. The use of FDG-PET in oncology to predict the treatment outcome early is well-recognized in many tumors, including seminoma (28–30). In a recent study of 75 patients with advanced seminoma treated with conventional chemotherapy, the negative FDG uptake after 2 cycles resulted in a prognostic factor suggesting its possible role in the chemotherapy de-escalation (29). This is to be evaluated in a prospective clinical trial. In our case, FDG-PET aided in evaluating the disease response after only two cycles of PEB therapy, but FDG-PET is not recommended in the re-staging of patients with non-seminomatous tumors after chemotherapy. Even this tool could be very helpful, no consensus data are available to consider it as standard tool to be routinely incorporated in clinical practice, but only in the clinical management of selected highly complex oncological cases where chemotherapy is not well-standardized.

Due to the rarity and heterogeneity of these cases, we are unable to conduct large clinical trials. Therefore, surgery remains a therapeutic approach that should always be considered in the management of TMT tumors and the choice of chemotherapy should be discussed in detail. A worldwide retrospective collection of cases could provide a larger dataset on chemotherapy treatments for such cases and enlarge the body of evidence on this rare setting.

The clinical findings described refer to that of a patient with a complete remission of disease 40-months after GCT-oriented therapy, who is now well and continues with the routine follow up with CT scan and CT-PET every 4 months. However, CT-PET is not routinely recommended for the follow up of testicular tumors as there is no evidence that it can provide additional advantage over CT (31).

In conclusion, with the limitation of being an isolated case, our case may suggest that germ cell tumor with somatic type malignancy of the sarcoma type could benefit more from a GCT-oriented therapy and not from sarcoma histology transformation therapy. The biological background explaining this outcome still remains unclear.

## Data Availability Statement

The data presented in this study are available on request by contacting Dr Valentina Indio valentina.indio2@unibo.it.

## Ethics Statement

Ethical review and approval was not required for the study on human participants in accordance with the local legislation and institutional requirements. The patients/participants provided their written informed consent to participate in this study.

## Author Contributions

MP, MM, AAr, and AAs: study concept and design. MU, AS, MF, TF, VA, VD, MI, SC, and AAl: acquisition of data. MP, MM, MN, LG, MS, and AAr: patient's clinical supervision. VI: data analysis. MP, MM, VI, MU, and AAs: drafting of the manuscript. MP and AAs: study supervision. All authors contributed to the article and approved the submitted version.

## Conflict of Interest

The authors declare that the research was conducted in the absence of any commercial or financial relationships that could be construed as a potential conflict of interest.

## Supplementary Material

Supplementary Table 1 Mutational events in the primary tumor and TMT relapse in sarcoma samples.
